# Physico-chemical and biological characterization of anopheline mosquito larval habitats (Diptera: Culicidae): implications for malaria control

**DOI:** 10.1186/1756-3305-6-320

**Published:** 2013-11-04

**Authors:** Seid Tiku Mereta, Delenasaw Yewhalaw, Pieter Boets, Abdulhakim Ahmed, Luc Duchateau, Niko Speybroeck, Sophie O Vanwambeke, Worku Legesse, Luc De Meester, Peter LM Goethals

**Affiliations:** 1 Department of Environmental Health Science and Technology, Jimma University, P.O. Box 378, Jimma, Ethiopia; 2 Department of Biology, Jimma University, Jimma, Ethiopia; 3 Laboratory of Environmental Toxicology and Aquatic Ecology, Ghent University, J. Plateaustraat 22, B-9000 Ghent, Belgium; 4 Department of Geography, Jimma University, Jimma, Ethiopia; 5 Department of Comparative Physiology and Biometrics, Ghent University, Ghent, Belgium; 6 Institute for Health and Society (IRSS), Université Catholique de Louvain, Brussels, Belgium; 7 Georges Lemaître Centre for Earth and Climate Research, Earth & Life Institute, Université Catholique de Louvain, Place Pasteur, 3, 1348 Louvain-la-Neuve, Belgium; 8 Department of Environmental Engineering, University of Connecticut, Storrs - Mansfield, USA; 9 Laboratory of Aquatic Ecology, Evolution and Conservation, University of Leuven, Ch. Deberiotstraat 32, B-3000 Leuven, Belgium

**Keywords:** Decision trees, Generalized linear model, Macroinvertebrate predators, Mosquito control, Mosquito larvae

## Abstract

**Background:**

A fundamental understanding of the spatial distribution and ecology of mosquito larvae is essential for effective vector control intervention strategies. In this study, data-driven decision tree models, generalized linear models and ordination analysis were used to identify the most important biotic and abiotic factors that affect the occurrence and abundance of mosquito larvae in Southwest Ethiopia.

**Methods:**

In total, 220 samples were taken at 180 sampling locations during the years 2010 and 2012. Sampling sites were characterized based on physical, chemical and biological attributes. The predictive performance of decision tree models was evaluated based on correctly classified instances (CCI), Cohen’s kappa statistic (κ) and the determination coefficient (R^2^). A conditional analysis was performed on the regression tree models to test the relation between key environmental and biological parameters and the abundance of mosquito larvae.

**Results:**

The decision tree model developed for anopheline larvae showed a good model performance (CCI = 84 ± 2%, and κ = 0.66 ± 0.04), indicating that the genus has clear habitat requirements. Anopheline mosquito larvae showed a widespread distribution and especially occurred in small human-made aquatic habitats. Water temperature, canopy cover, emergent vegetation cover, and presence of predators and competitors were found to be the main variables determining the abundance and distribution of anopheline larvae. In contrast, anopheline mosquito larvae were found to be less prominently present in permanent larval habitats. This could be attributed to the high abundance and diversity of natural predators and competitors suppressing the mosquito population densities.

**Conclusions:**

The findings of this study suggest that targeting smaller human-made aquatic habitats could result in effective larval control of anopheline mosquitoes in the study area. Controlling the occurrence of mosquito larvae via drainage of permanent wetlands may not be a good management strategy as it negatively affects the occurrence and abundance of mosquito predators and competitors and promotes an increase in anopheline population densities.

## Background

Mosquitoes are not only a nuisance, but are also responsible for the spread of a wide range of diseases including malaria, yellow fever, dengue, West Nile virus and Rift Valley fever [1-3]. These mosquito borne diseases, infecting more than 700 million people around the world each year, result in as many as two million deaths annually [4]. One of these diseases, malaria, is transmitted between humans by adult female mosquitoes of the genus *Anopheles.* Malaria is endemic in tropical and sub-tropical regions where it causes over 300 million acute illnesses and at least one million deaths each year [5]. In spite of the recent scale-up of control programs, malaria continues to be a major public health problem in most tropical countries and its control is becoming increasingly difficult due to the spread of resistance of the parasite to anti-malarial drugs, resistance of the vector to insecticides and land-use changes [6,7].

Land-use and land-cover changes, such as deforestation, agricultural expansion, infrastructure development, urbanization and human population growth contribute to the proliferation of breeding sites of mosquitoes [5,8]. These environmental or land-use modifications also affect climate processes [9] that are likely to support rapid development of mosquitoes and parasites in regions where there has previously been a low-temperature restriction on transmission. Current episodes of climate variability in Africa are likely to intensify the transmission of malaria in the eastern and southern highlands [10,11]. Moreover, dams and small irrigation projects also contribute to an increase in the mosquito population by, increasing the number of suitable larval habitats, prolonging the breeding season and allowing the expansion of their distribution range. Small dams built for irrigation and mega hydropower dams have been shown to favour malaria transmission in Ethiopia due to habitat creation [12,13].

Several studies have examined the relationship between habitat characteristics and mosquito larval abundance and distribution in Africa [14-18]. *Anopheles arabiensis*, the principal malaria vector in Sub-Saharan Africa, prefers shallow clean water and sunlit temporary habitats such as sand pools, brick pits and rain pools [15,16]. The presence of *An. arabiensis* immature stages in aquatic habitats is mainly influenced by water temperature, emergent plant cover, water current, turbidity, canopy cover, substrate type, and presence of predators and competitors [15-17]. Shililu *et al.*[15] indicated that in low-and highlands in Eritrea, water temperature was positively correlated with larval density. Higher temperatures encourage better development of eggs or allow the development of more microorganisms that are used as food by the larvae [14]. On the other hand, high emergent plant cover of aquatic habitats is likely to reduce mosquito larvae by obstructing gravid females from ovipositing and supporting a high diversity of predators [17]. The occurrence of predators and competitors is also a key determinant for the presence of *An. arabiensis* larvae. Muturi *et al.*[17] indicated that gravid females of *An. arabiensis* would avoid ovipositing in habitats where members of the family Heptageniidae are present, presumably to avoid direct competition. Furthermore, *An. arabiensis* is virtually absent or present at low abundance in habitats where there are predators such as fish (Tilapia, *Oreochromis sp*.), dragonfly larvae, water bugs and water beetles [19].

Malaria vector control has been largely dependent on the use of chemical insecticides. Only 12 insecticides belonging to four insecticide classes are recommended for public health use either for indoor residual spraying or to treat mosquito nets [20]. Unfortunately, resistance to insecticides has been reported from many malaria vector species. Resistance spreads rapidly, which constitutes a serious threat to malaria control initiatives [20]. In Ethiopia, populations of *An. arabiensis*, the major malaria vector in the country, developed resistance to three (organochlorines, organophosphates and pyrethroids) out of the four insecticide families commonly used for public health use [21,22]. Therefore, alternative malaria vector control tools, targeting mosquito immatures either alone or as part of integrated vector management, should be envisaged to reduce human-vector contact and hence malaria transmission intensity.

Adult mosquitoes are difficult to control since they can fly relatively long distances and survive in a wide range of microhabitats, including the soil and in holes in rocks and trees [23]. Effective mosquito larval control can be achieved through larval habitat management [14,24]. Larval control through environmental management has gained a lot of attention during the last decades [25,26]. Environmental management involves changes in potential mosquito breeding areas to prevent, eliminate or reduce the vector’s habitat [26]. Techniques include draining man-made and natural wetlands, land levelling, filling small ponds or water collecting depressions and changing banks of water impoundments [25]. However, draining natural water bodies such as wetlands may affect the composition and structure of mosquito predators and species diversity in general more than they do reduce mosquito breeding sites [27]. Even after a wetland has been drained, it may often still hold enough water after a rain event to serve as a breeding site for mosquitoes [28]. In addition, drainage of wetlands often reduces important regulating ecosystem services such as mitigating floods, recharging aquifers, micro-climate stabilization and improving water quality [29]. So, draining wetlands does not seem to be a good strategy to reduce the habitat of mosquito vectors.

In order to include mosquito larval habitat management as part of an integrated vector management program, detailed knowledge on the ecology of the aquatic immature stages is crucial [30]. To this end, habitat suitability modelling has been increasingly used to determine the presence of malaria vectors and estimating their population levels. Such information is the basis for risk assessment of mosquito-borne diseases [31,32]. Habitat suitability models take into consideration the occurrence and/or abundance of species in relation to biotic and abiotic environmental factors, evaluating the habitat quality or predicting its effect on species occurrences as a result of environmental changes within the habitat [33]. However, species-habitat relationships are influenced by regional conditions and hence, the generality of these models needs to be tested [34]. Therefore, we developed data-driven models using decision trees and generalized linear models in order to assess the relationship between abiotic and biotic environmental factors and the occurrence and abundance of anopheline mosquito larvae in Southwest Ethiopia. This could help decision makers to identify priority habitats to be targeted for the control of anopheline mosquito larvae. We specifically addressed the question of whether permanent marshlands in the neighbourhood of Jimma (the main city in the Gilgel Gibe catchment), which are bio-diverse areas that are under serious threat by land encroachment and which are perceived as mosquito breeding grounds, are indeed a preferred habitat for anopheline mosquito larvae. These marshlands fulfil many ecosystem services so their destruction would entail important losses and a good and integrated management is therefore required.

## Methods

### Study area

**Figure 1:**
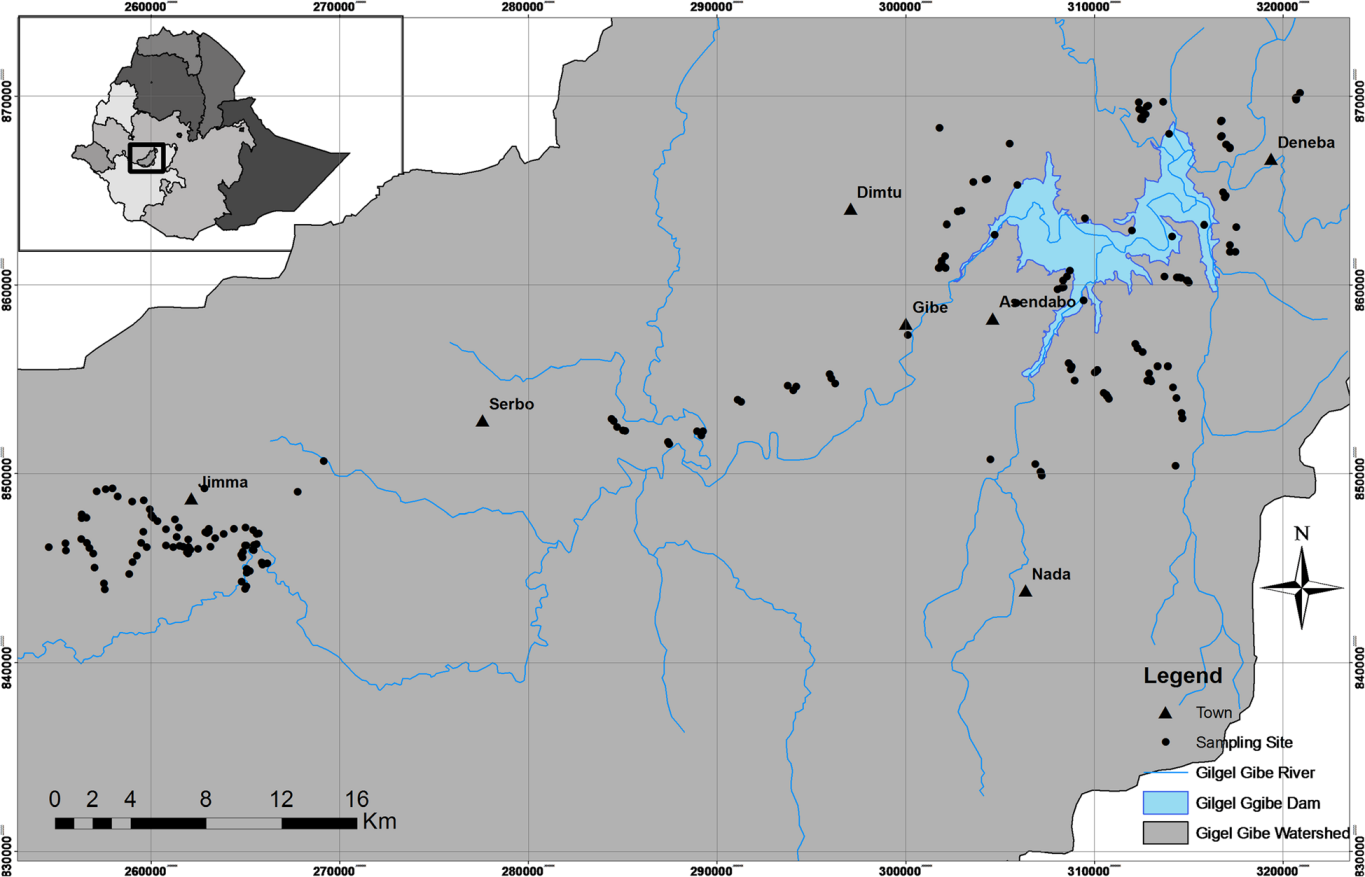
Map of the study area with indication of the sampling sites in the Gilgel Gibe I watershed, Southwest Ethiopia.

This study was conducted in the Gilgel Gibe I watershed situated in Southwest Ethiopia, lying between latitudes 7°37’N and 7°53’N and longitudes 36°46’E and 37°43’E (Figure 1). The elevation of the study area ranges from 1,650 to 1,800 meters above sea level. The mean annual temperature in the area is between 15°C and 22°C, and the mean annual precipitation is between 1800 mm and 2300 mm, with maximum rainfall from June till early September and minimum precipitation between December and January [35]. The study area is characterized by different land use patterns. The main socio-economic activities of the inhabitants are farming and small stock rearing, with maize (*Zea mays*) and teff (*Eragrostis tef)* being the main crops cultivated in the area. The region is, however, also known for its coffee production. The average population density in this area is approximately 100 to 110 people/km^2^.

### Characterization of larval habitats

**Figure 2:**
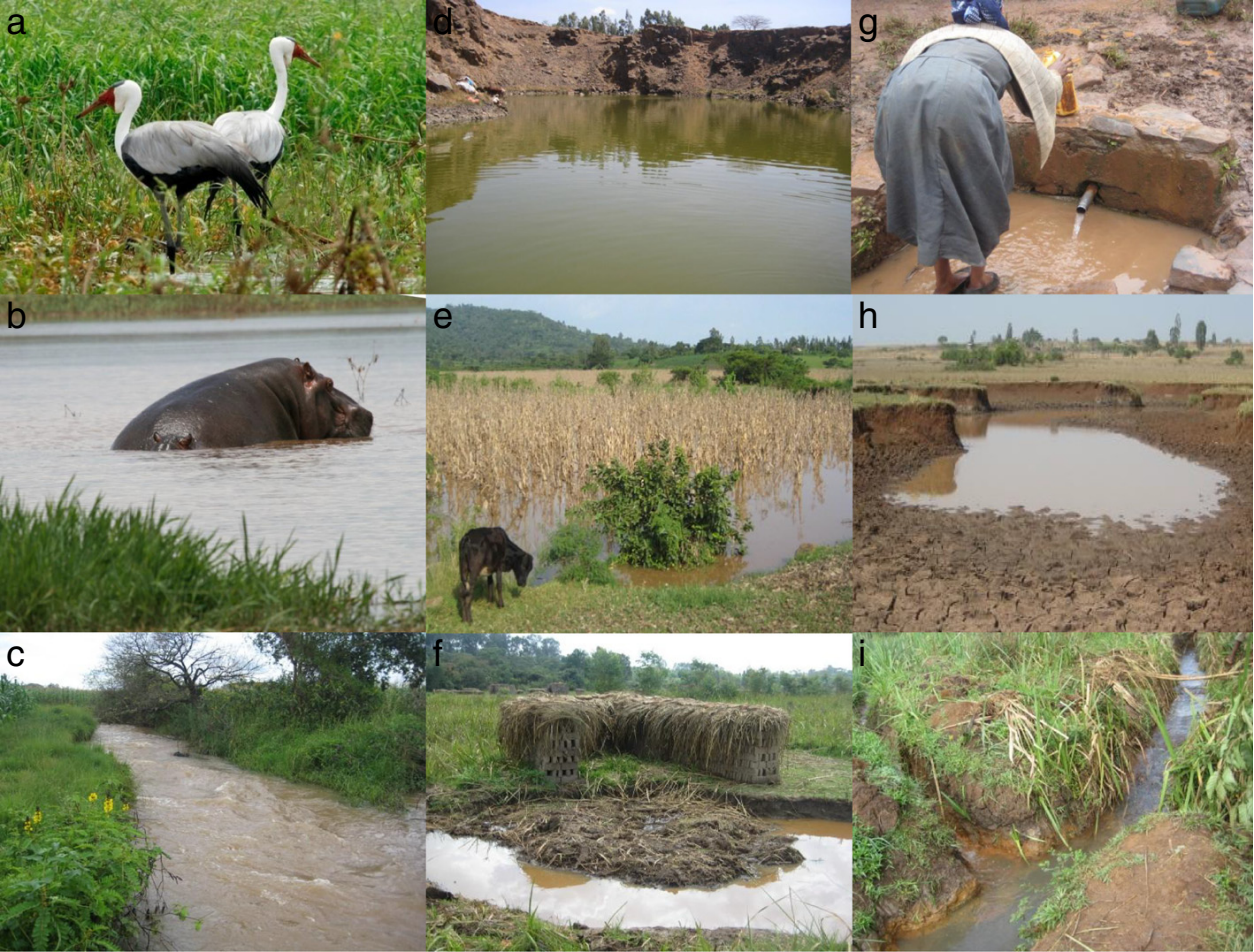
Pictures of Different habitat types sampled in the studied wetlands: natural vegetated wetland (a), natural open water wetland (b), stream fringe (c), pond (d), maize field (e), brick pit (f), pool (g), rain pool (h), drainage ditch (i).

A total of 220 samples were taken at 180 different sampling locations (larval habitats) between August and October 2010 and September to November 2012. Selection of surveyed sites was based on previous reports on surface water quality monitoring [36] and distribution of disease vectors in the region [22]. Sampling sites situated in permanent habitats such as natural wetlands, reservoir and streams were selected along a gradient of visible disturbance including point source pollution, land use pattern, hydrological modification and accessibility. Sampling sites situated in temporary breeding habitats were randomly selected from six villages located up to 8 km from the Gilgel-Gibe hydroelectric dam and from temporary pools located around permanent habitats. Permanent habitats were sampled at exactly the same location during both years, while the sampling location of temporary habitats changed depending on the availability of water. Temporary habitats are those containing water for a short period of time (approximately two weeks after the end of rainy season). Semi-permanent habitats are those containing water for 2 to 3 months after the rainy season ends. Permanent habitats are those containing water throughout the year (fed by surface or ground water) and are more stable systems. Surveyed habitats included: natural wetlands (n = 60), breeding habitats around the shore of the dam reservoir (n = 13), natural ponds (n = 10), streamed pools (n = 30), farm ditches (n = 25), pits for plastering (n = 40), rain pools (n = 20), vehicle ruts (n = 12) and animal hoof prints (n = 10) (Figure 2). Detailed information on habitat condition, water quality, presence of anopheline larvae and mosquito predators and competitors was collected during the survey.

Data on size of the water body (area), substrate type, vegetation cover, canopy cover and land use pattern were collected for each larval habitat. Water depth was measured using a metal ruler at different points of each habitat and average depth was recorded. Substrate was classified into clay, silt, sandy, gravel and artificial substrate (concrete, tire, plastic and mud pot). The emergent, submerged and floating plant cover of a habitat was visually estimated as the percentage cover of these aquatic macrophytes within a 500 metre stretch for large aquatic habitats and the entire area for smaller habitats. Plant cover was categorized as very low (<10%), low (10-35%), moderate (35-65%), high (65-90%) and very high (>90%) [37]. Canopy cover was defined as the amount of vegetation covering the water surface. Canopy within or the surrounding of the sampling site was estimated visually based on the percentage of shade [38]. The type of land use adjacent to each sampling site was also recorded and checked with the available GIS data on land use. The map templates including land use types were obtained from the Ethiopian Ministry of Water and Energy.

Habitat characterization, including dissolved oxygen, conductivity, pH and water temperature were measured using a multi-probe meter (HQ30d Single-Input Multi-Parameter Digital Meter, Hach). A hand-held hygrometer (RH87) was used to measure ambient air temperature and relative humidity. Turbidity was measured using AquaFluor Handheld Fluorometer/Turbidimeter. Water chemistry analysis was carried out by sampling 2 l of water from each sampling site. The water sample was stored in an icebox and transported to the Laboratory of Environmental Health Science and Technology, Jimma University. The samples were then analysed for total dissolved solids (TDS), alkalinity, hardness, chloride, and orthophosphate and nitrate concentration following standard methods [39].

Geographic coordinate readings were recorded for all sampling sites using a hand-held global positioning system unit (GPS) (Garmin GPS 60, Garmin international Inc., and Olathe, Kansas, USA). Coordinate readings were integrated into a GIS database using Arc MAP 10 GIS software. All digital data in the GIS were displayed in the World Geodetic System (WGS) 1984 Coordinate system.

### Mosquito larvae sampling and identification

To collect mosquito larvae, one to ten dip samples were taken from each habitat using a standard 350 ml dipper (Clarke Mosquito Control Products, Roselle, IL) depending on the habitat size. Mosquito larvae were also sampled using 5 ml graduated pipettes from water bodies, which were too small to use standard dippers. For small habitats such as hoof prints, several hoof prints were pooled to get the required sample volume. Quantitative sampling from small habitats may overestimate larval density as compared to large habitats since larvae may not escape in small habitats where whole water can be sampled [40]. The use of different sampling methods may affect the analysis of abundance data, which could be considered as a limitation of the study. Water collected by dippers was emptied into a white enamel sorting tray and mosquito larvae were sorted and identified to genus level as either anopheline or culicine. The presence of mosquito immature stages was defined by the presence of at least one larva or pupa found in any of the ten dips.

### Mosquito predator and competitor sampling and identification

A rectangular frame net (30 × 20 cm) with a mesh size of 250 μm was used to sample mosquito predators and competitors at the same sampling sites where mosquito larvae sampling was carried out. Each collection entailed a 10 minute kick-sample with a hand net over a distance of 10 metres in the habitats that were sufficiently large [41]. Time was allotted proportionally to the percentage cover of different mesohabitats (i.e., bottom, mid-water, surface, and near debris). Small habitats (e.g. farm ditches, road puddles and pits) that could not be sampled by kick-net were sampled using sweep nets. Contents collected in the sweep or kick-net were emptied on a white sorting tray to enhance visibility and counting of the sampled organisms. Fish and tadpoles were recorded and released at their site of capture. Macroinvertebrates were sorted in the field, kept in vials containing 75% ethanol for later identification and enumeration. Macroinvertebrates were identified to family level in the laboratory using a stereomicroscope (10 × magnifications) and standard identification key [42]. Each family was categorized into one of the five functional feeding groups (FFG): gatherer-collector, filterer-collector, predator, scraper, and shredder [43]. When multiple possible FFGs were identified for a particular family, the most commonly occurring classification was used. All identified macroinvertebrates, their frequency of occurrence in the study area and their FFG are presented (Additional file 1). Filter-collectors such as tadpole, black fly (Simuliidae), bivalve molluscs (Sphaeriidae) caddisfly larvae (Hydropsychidae) and culicine larvae were considered as competitors of anopheline larvae [44]. Fish and aquatic invertebrates belonging to the orders Hemiptera (water bugs), Coleoptera (Water beetles) and Odonata (dragonflies and damselflies) were considered as predators [44]. Presence or absence (1/0) of invertebrate predators and competitors were used as independent variables in the classification tree models.

### Data analysis

Twenty five input variables were used to identify the main predictors of mosquito larvae occurrence and abundance (Table 1). We used classification and regression tree (CART) models and ordination analysis to investigate the relationship between anopheline mosquito larvae occurrence and abundance and different explanatory variables. In addition, occurrence and abundance of anopheline larvae were analysed using logistic and Poisson regression models (Additional file 2 and Additional file 3). CART analysis is a form of binary recursive partitioning that can be used to classify observations [45]. It has a number of advantages over traditional generalized linear models. First, it is well suited for analysis of complex ecological data with high–order interactions [45,46]. Second, it captures non-linear relationship between explanatory and response variables [46]. Third, it does not rely on the assumptions that are required for parametric statistics and the analysis is not restricted by multicollinearity in predictor variables [47]. Fourth, missing values are not dropped from the analysis, instead variables containing information similar to that contained in the primary splitter are used [47]. CART trees are also relatively simple for non-statistician to interpret [47]. However, CART may produce different models depending on the selection of input variables [48]. Ordination methods are widely used for community analysis [49], and typically assume that abundance of individual species vary in a linear or uni-modal manner along environmental gradients [50].

**Table 1:** Input variables used for habitat preference analysis with their mean, standard deviation (SD) and minimum and maximum values (range)

**Variables**	**Unit**	**Mean**	**SD**	**Range**
**Altitude**	Meter above sea level	1725	35	1655–1800
**Area**	Hectare	0.65	0.9	0–7.8
**Water depth**	Meter	0.37	0.2	0–1.42
**Canopy cover**	%	6	15	0–100
**Air temperature**	°C	27	3	19–39
**Water temperature**	°C	24	3	16–34
**pH**	-	7	0.6	5.4–10
**Dissolved oxygen**	mg/l	4.7	2	0.47–10
**Conductivity**	μS/cm	112	55	21–513
**Total dissolved solid**	mg/l	106	77	15–425
**Turbidity**	NTU	160	218	4–894
**Alkalinity**	mg/l	58	33	0–250
**Hardness**	mg/l	37	23	0–160
**Nitrate**	mg/l	0.4	0.48	0–2.3
**Ortho-phosphate**	mg/l	0.12	0.2	0–1.4
**Permanency**	Temporary(1), semi-permanent(2), Permanent(3)	N/A	N/A	N/A
**Emergent plant cover**	Very low to very high	2	1.6	0–4
	5 class (0–4)			
**Submerged plant**	Very low to very high	0	0.5	0–4
	5 class (0–4)			
**Floating plant**	Very low to very high	0	0.5	0–4
	5 class (0–4)			
**Habitat type**	9 types (see Table 2)	N/A	N/A	N/A
**Substrate type**	Silt(1), sandy(2), gravel(3), artificial substrate(4)	N/A	N/A	N/A
**Land-use**	9 types (See Figure 3)	N/A	N/A	N/A
**Fish**	Absence(0), presence(1)	N/A	N/A	N/A
**Invertebrate predators**	Abundance	28	42	0–232
**Competitors**	Abundance	2.7	4	0–23

*N/A* not applicable.

### Classification and regression tree models (CART)

Classification tree (CT) models were used to model the occurrence (presence/absence) of anopheline larvae based on measured environmental factors. The CT models were built using the J48 algorithm [51], a java re-implementation of the C4.5 algorithm, which is a part of machine learning package WEKA [52]. Likewise, regression tree (RT) models were used to model the abundance of anopheline larvae [52]. The RT models were built using the M5 algorithm in WEKA [51]. Regression tree models have been previously successfully used in malaria studies [53]. Default parameter settings were used to induce the decision trees. Model training and validation were based on a three-fold cross-validation procedure [51]. The dataset was randomly shuffled into three equal subsets and each subset in turn was used for validation, while the remaining two subsets were used for training. The cross-validation process was then repeated three times each time with one of the three subsets used as the validation dataset. The predictive performance (based on the percentage of correctly classified instances and Cohen’s kappa statistic) of the subsets were averaged to produce a single prediction of the dependent variable. The variation was also assessed based on the difference between the outcomes of the subsets.

The mean percentage of correctly classified instances (CCI) [51] and Cohen’s Kappa statistic (κ) [54] were used to evaluate the predictive performance of the classification tree model. The CCI is the percentage of the true positive (TP) and true negative (TN) predictions, whereas Cohen’s Kappa statistic simply measures the proportion of all possible cases of presences or absences that are predicted correctly by a model, accounting for chance effects. Models with a CCI higher than or equal to 70% and κ higher than or equal to 0.4 were considered reliable [55]. CCI is affected by the frequency of occurrence of the taxon being modelled [55]. Unlike CCI, κ takes a correction into account for the expected number of correct predictions due to randomness, which is strongly related to taxon prevalence [55]. We used the following ranges of κ recommended by [55] for model performance evaluation: poor (κ = 0), slight (κ = 0–0.2), fair (0.2–0.4), moderate (κ = 0.4–0.6), good (κ = 0.6–0.8) and nearly perfect (κ = 0.8–1). We used the determination coefficient (R^2^) value to evaluate the performance of the regression tree models [46]. The closer the value to one, the better the model performed.

A conditional analysis was performed in order to see how different values of a predictor variable influence the abundance of anopheline larvae. For each of the three regression tree submodels developed (based on the three folds), the influence of predictor variables on the abundance of anopheline larvae was analysed. Regression equations obtained from the submodels were then used to calculate the abundance of anopheline larvae. This was achieved by taking minimum and maximum values of the predictor variables, while other variables, which were present in the model were kept constant at average values. Hence, for each of the three different subsets (folds) a line was plotted showing the relationship between the predictor variables and the abundance of anopheline larvae.

### Ordination analysis

To determine whether a linear or unimodal type of response was present along environmental gradients, the data-set was first analysed using a detrended correspondence analysis (DCA) in CANOCO for Windows version 4.5 [56]. Redundancy analysis (RDA) was then used because all environmental gradients were shorter than 2 standard deviation units. In all RDA analyses, the abundance of anopheline larvae, predators and competitors were considered as response variables, whereas environmental variables were treated as independent variables. A preliminary analysis was performed to test multi-collinearity in environmental variables. When two or more variables had a variance inflation factor of greater than 5, one of these variables was removed from the analysis.

Based on a stepwise forward selection, twelve environmental factors were selected as independent variables. Species and environmental data, except for pH, were log transformed [log(x + 1)] prior to analysis to stabilize the variance. The statistical significance of eigenvalues and species-environment correlations generated by the RDA were tested using Monte-Carlo permutations.

### Analysis of abundance of mosquito predators and competitors in different habitat types

We made Box-and Whisker plots in STATISTICA 7.0 [57] to visualize the abundance of mosquito predators and competitors in different habitat types. Abundance data were log transformed [log(x + 1)] prior to analysis. We used a non-parametric, Kruskal-Wallis test at a significance level of 0.05, to determine whether significant differences in the abundance of invertebrate predators and competitors existed between different habitat types.

## Results

### Occurrence and distribution of mosquito larvae

**Figure 3:**
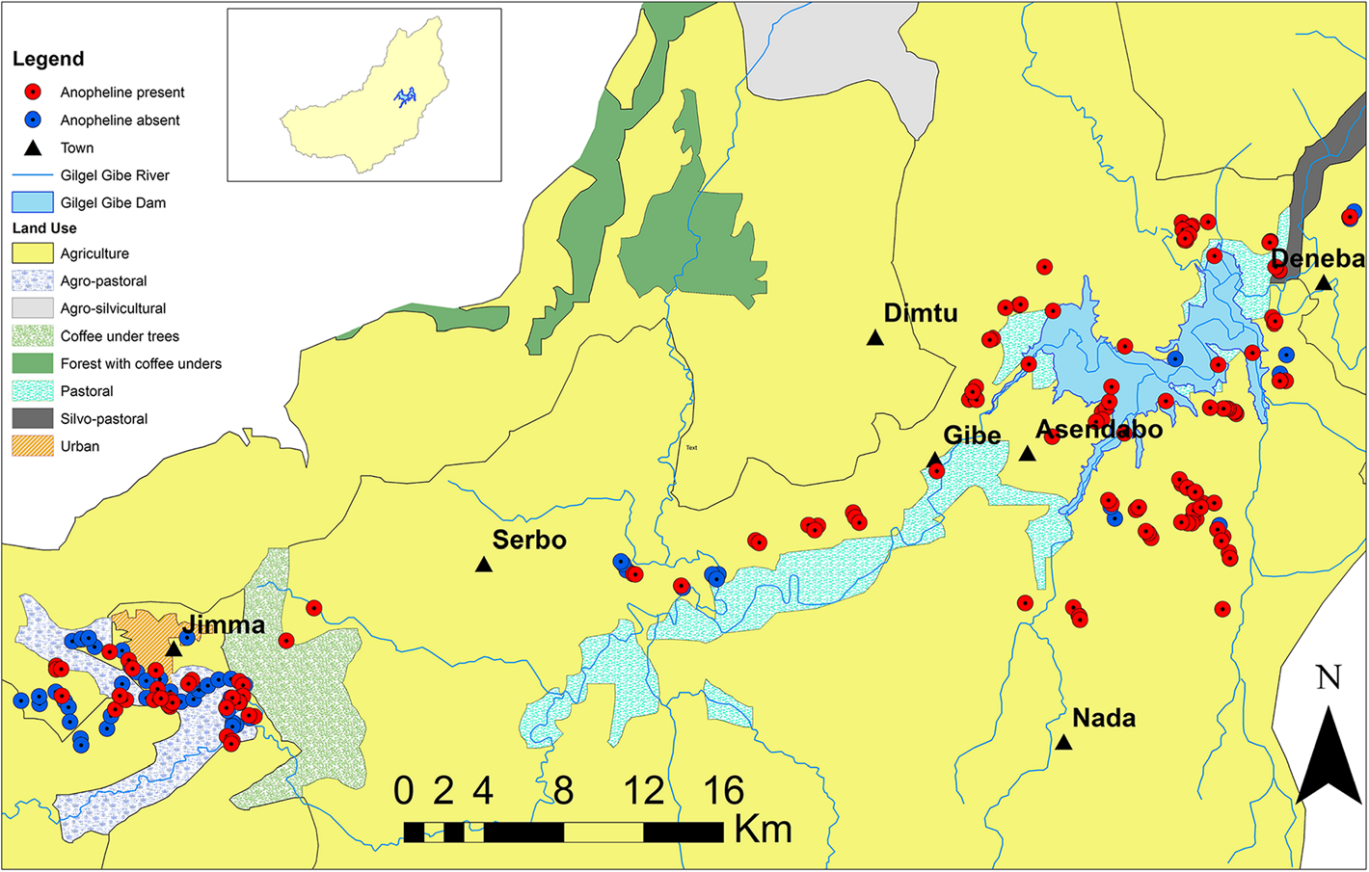
Map showing the distribution (presence (blue) and absence (red)) of anopheline larvae in the Gilgel Gibe I sub-catchment, Southwest Ethiopia.

A total of 220 samples were collected from 180 sampling sites. Anopheline larvae occurred more frequently in pits dug for plastering, vehicle ruts and farm ditches and less frequently in natural wetlands and ponds (Table 2). Overall, 1220 anopheline larvae individuals were found in 151 samples (69% frequency of occurrence). A total of 496 culicine larvae individuals were found in 62 samples (28% frequency of occurrence). The anopheline positive habitats were mainly located in agricultural and agro-pastoral land use types (Figure 3). Anopheline larvae were sparsely distributed in natural wetlands.

**Table 2:** Distribution of anopheline larvae among different larval habitat types, Southwest Ethiopia

**Habitat type**	**No. of samples**	**Anopheline positive samples n (%)**
	**N = 220**	
**Marshland**	60	24(40)
**Reservoir**	13	7(54)
**Farm ditch**	25	23(92)
**Pond**	10	5(50)
**Road puddle**	12	11(92)
**Stream margin**	30	17(57)
**Rain pool**	20	17(85)
**Pit**	40	38(95)
**Hoof print**	10	9(90)

### Influence of environmental factors on the occurrence of anopheline mosquito larvae

**Figure 4:**
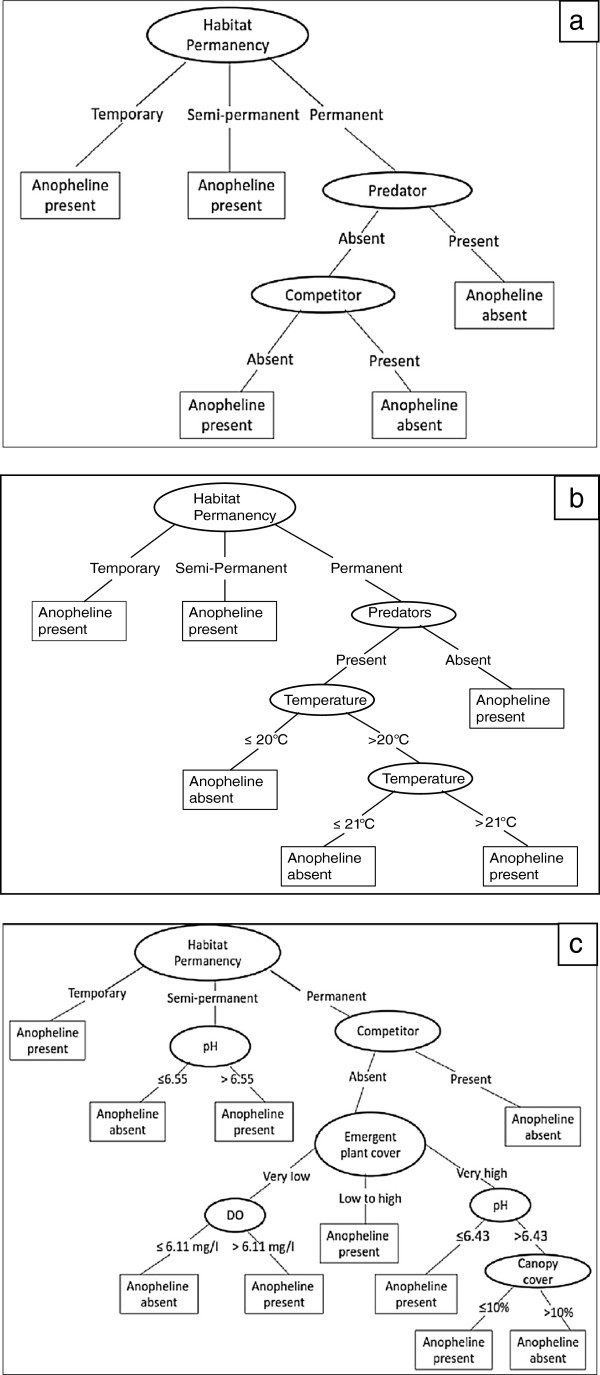
**Classification tree model assessing the presence or absence of anopheline larvae. (a)** subset one (Correctly Classified Instances = 86%, Cohens kappa statistic = 0.63), **(b)** subset two (Correctly Classified Instances = 82.4%, Cohens kappa statistic = 0.63), **(c)** subset three (Correctly Classified Instances = 86.5%, Cohens kappa statistic = 0.71).

Based on the three models (one model for each fold or subset) developed, the most frequently selected variables were habitat permanency (100%) and occurrence of predators and competitors (67%). Moreover, habitat permanency was selected as the root of the tree for all models, indicating that this was the most important variable determining the presence/absence of anopheline larvae. The classification tree of subset one (Figure 4a) has five leaves and eight branches. Habitat permanency was selected as root of the tree. Anopheline larvae were present in both temporary and semi-permanent habitats. In contrast, anopheline larvae were absent in permanent habitats when predators or competitors were present. This classification tree model had a good predictive performance, with a CCI of 86% and κ of 0.63. The classification tree model based on subset two (Figure 4b) has six leaves and ten branches. Similar to subset 1, habitat permanency was selected as a root of this tree. Anopheline larvae were present in both temporary and semi-permanent habitats. In contrast, anopheline larvae were absent in permanent habitats when predators were present and water temperature was less than 20°C. This classification tree model had a good predictive performance, with a CCI of 82.4% and κ of 0.63. The classification tree model based on subset three (Figure 4c) has twelve leaves and nineteen branches. Habitat permanency was selected again as root of the tree. Anopheline larvae were present in temporary habitats. The occurrence of anopheline larvae in permanent habitats was influenced by several biotic and abiotic factors. This classification tree model had a very good predictive performance, with a CCI of 86.5% and κ of 0.71. The importance of biotic factors such as invertebrate predators and competitors and abiotic factors such as permanency on the occurrence of anopheline larvae was also indicated by Generalize Linear Models (GLMs) (See Additional file 2).

### Influence of environmental factors on the abundance of anopheline mosquito larvae

**Figure 5:**
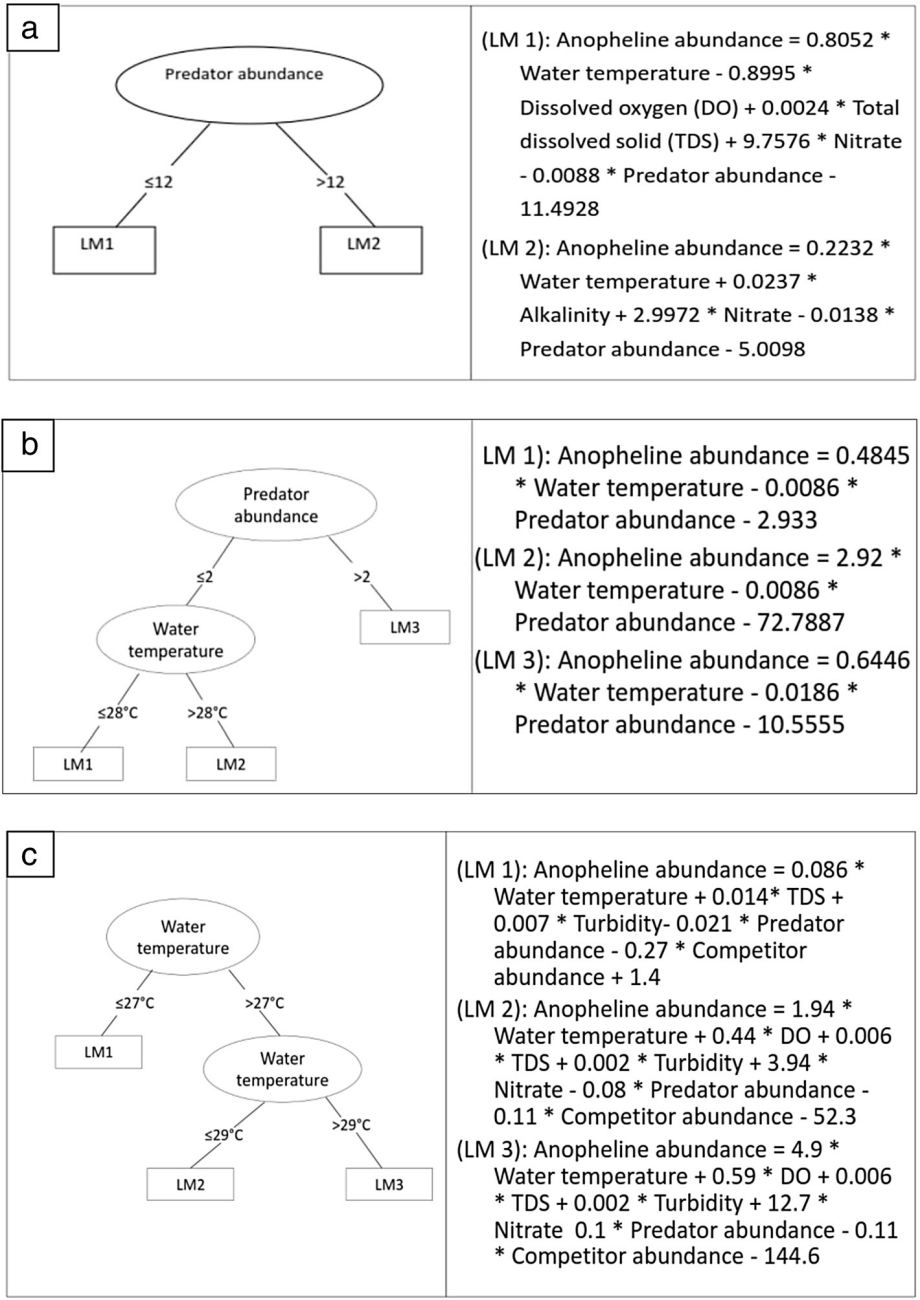
**Regression tree with regression equations predicting the abundance of anopheline larvae. (a)** subset 1 (Determination coefficient = 0.39), **(b)** subset 2 (Determination coefficient = 0.44), **(c)** subset 3 (Determination coefficient = 0.42).

The regression tree model based on subset one predicting the abundance of anopheline larvae has a determination coefficient of 0.44. If the abundance of predators was less than or equal to 12 individuals per sample, LM1 was applied, in case the abundance was higher than 12 individuals, LM2 was used (Figure 5a). According to LM1, the abundance of anopheline larvae increased with increasing water temperature, total dissolved solids, nitrate concentration and decreased with increasing predator abundance and dissolved oxygen concentration. For LM2, the abundance of anopheline larvae increased with increasing water temperature, alkalinity and nitrate and decreased with increasing abundance of predators. The regression tree model based on subset two has three leaves and a determination coefficient of 0.44 (Figure 5b). If the abundance of predators was lower than 2 individuals per sample and water temperature was lower than 28°C, LM1 was applied. In case the temperature was higher than 28 LM2 was applied, whereas if the abundance of predators was higher than 2, LM3 was used (Figure 5b). The regression tree model indicated that the abundance of anopheline larvae increased with increasing water temperature and decreased with increasing predator abundance. The regression tree model based on subset three has three leaves and a determination coefficient of 0.42 (Figure 5c). If water temperature was lower than or equal to 27°C, the linear model LM1 was applied. In case temperature was between 27-29°C LM2 was applied, whereas when temperature was higher than 29°C LM3 was applied. According to the model the abundance of anopheline larvae increased with increasing water temperature, total dissolved solids and turbidity and decreased with increasing predator and competitor abundance.

**Figure 6:**
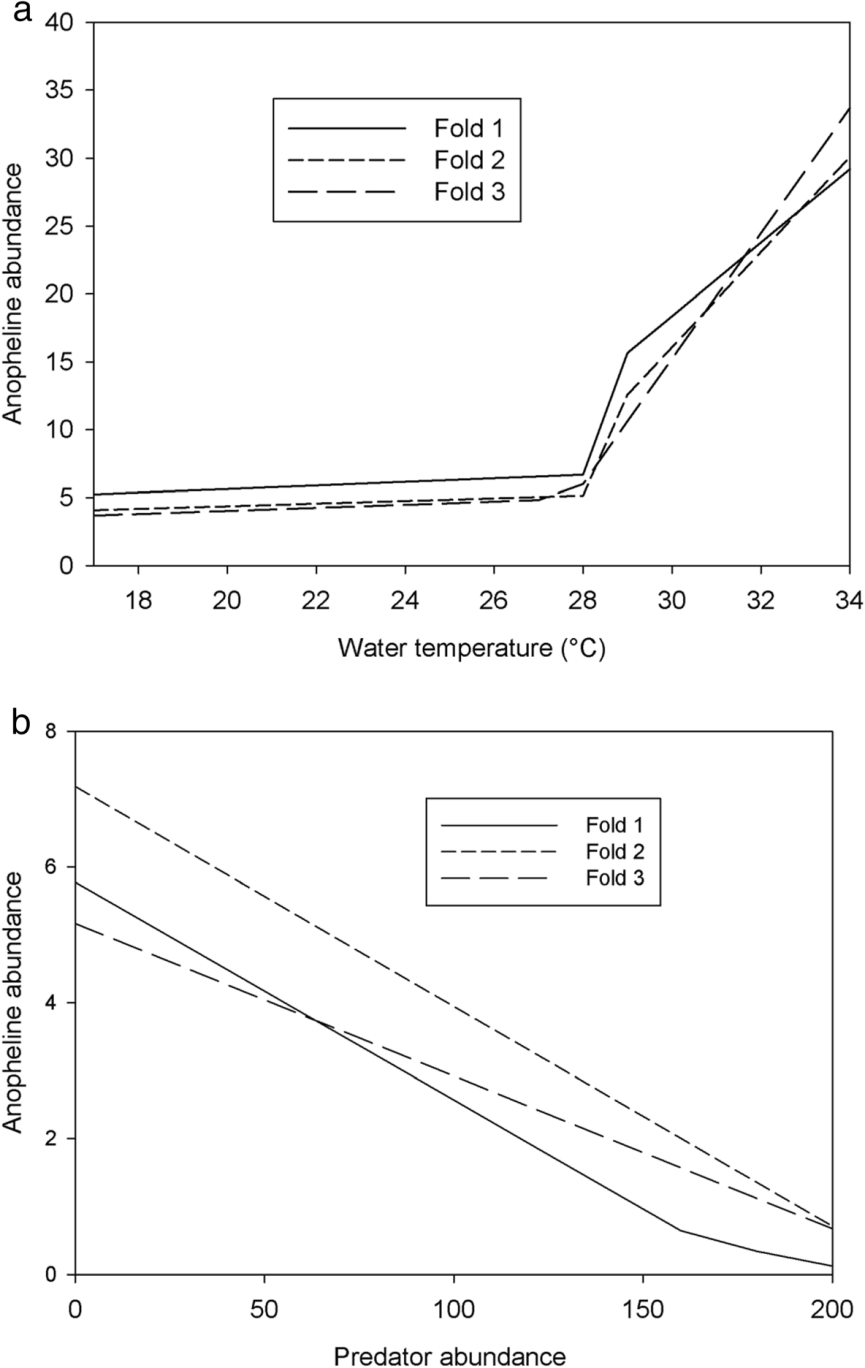
Conditional analysis visualized for the abundance (number of individuals per sample) of anopheline larvae in function of (a) water temperature; (b) abundance of macroinvertebrate predators.

A conditional analysis of the regression tree model (all 3subsets) showing the effect of water temperature on the abundance of anopheline larvae is shown in Figure 6a. A slight increase in anopheline larval abundance was noted at a temperature between 17°C and 28°C, whereas an abrupt increase was observed between 28°C and 34°C. On the other hand, the abundance of anopheline larvae declined with increasing abundance of macroinvertebrate predators (Figure 6b). The importance of water temperature on the abundance of anopheline larvae was also indicated by GLMs (see Additional file 3).

**Figure 7:**
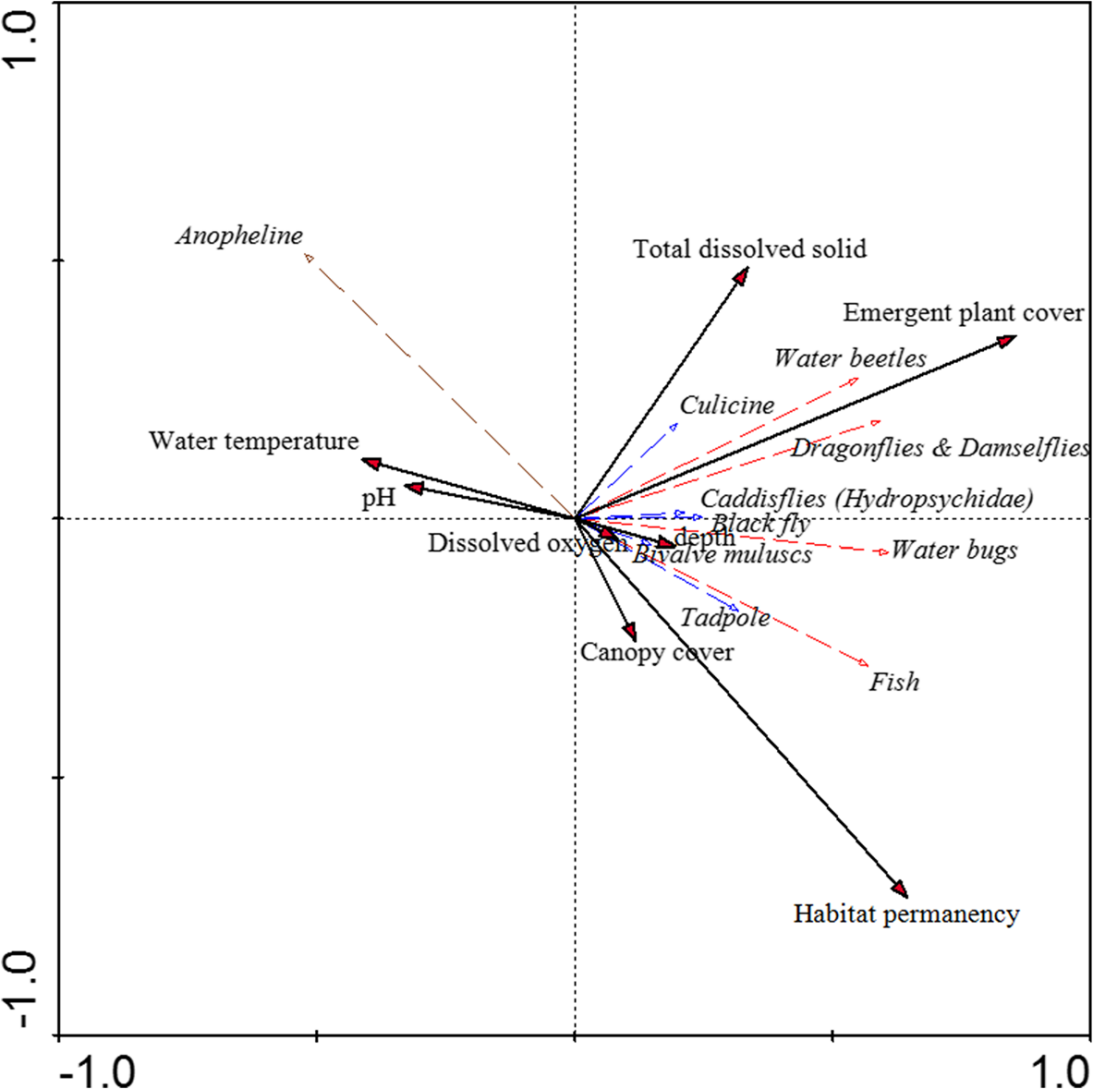
**Ordination plot of anopheline larvae and environmental and biological variables based on the redundancy analysis (RDA).** Competitors of anopheline larvae are indicated by blue arrows and predators of anopheline larvae by red arrows.

The detrended correspondence analysis (DCA) gave a length of gradient smaller than 2 standard deviation units, implying that anopheline larvae exhibit a linear response to environmental gradients [58]. The association between anopheline larvae and the selected environmental variables was found to be significant (p < 0.05) for both the first axis and all canonical axes together (Figure 7). The variance of the RDA-biplot of anopheline larvae and environmental variables based on the first two axes explained 33% of the variance in anopheline data and 94% of the variance in the correlated and class means of anopheline larvae with respect to the environmental variables. The eigenvalues of the first two axes were 0.27 and 0.06, respectively. In this ordination, the anopheline larvae-environment correlation for the first two axes was 0.77 and 0.67, respectively. The first axis of the RDA ordination revealed a gradient primarily associated with habitat permanency. This axis was negatively correlated with the occurrence of anopheline larvae (r = -0.8, p < 0.05). The second canonical axis described the emergent plants and mosquito predators and TDS gradient.

### Relationship between the abundance of mosquito predators and competitors and habitat types

**Figure 8:**
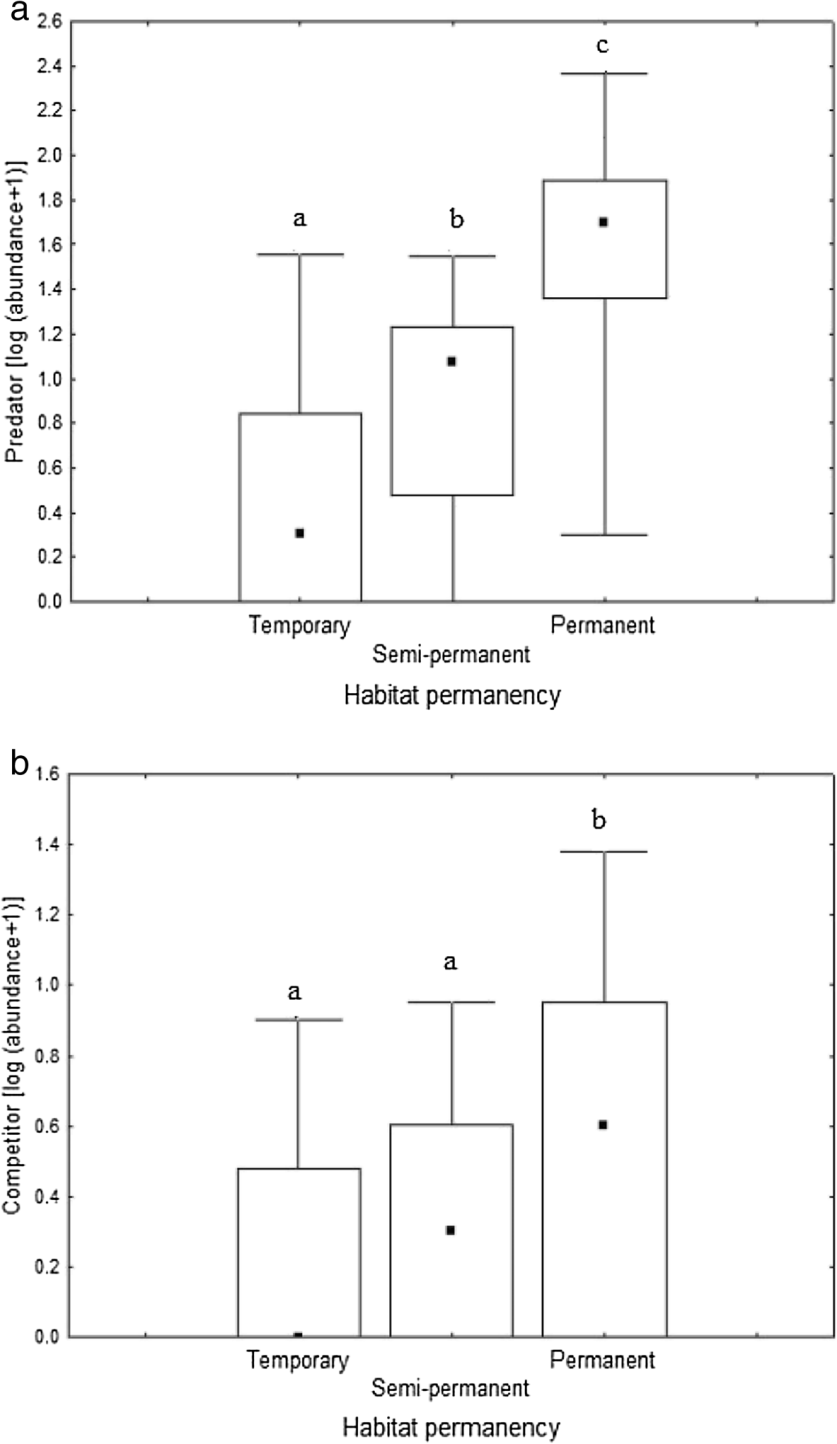
**Logarithmic abundance of predators and competitors in relation to habitat permanency.** Box- and Whisker plots of the log (abundance + 1) of predators **(a)** and competitors **(b)** in relation to habitat permanency. Small black squares represent median numbers, boxes represent interquartile ranges (25–75% percentiles) and range bars show maximum and minimum values. Statistically significant differences shown by Kruskal-Wallis test (p < 0.05) are indicated by letters.

Box- and Whisker-plots indicated that, there was a statistically significant difference in the abundance of invertebrate predators (*χ*2 = 93.2, df = 2, *p* <0.05) and competitors (*χ*2 = 15.9, df = 2, *p* < 0.05) among different habitat types (Figure 8). Permanent habitats support a significantly higher abundance of macroinvertebrate predators and competitors than temporary habitats (P < 0.05).

## Discussion

A fundamental understanding of the ecology of anopheline mosquito larvae is important in order to plan and implement effective malaria vector control intervention strategies [19]. In the present study, habitat permanency, canopy cover, emergent plant cover and occurrence and abundance of predators and competitors were found to be the main variables determining the abundance and distribution of anopheline larvae in aquatic habitats.

Temporary water bodies such as farm ditches, rain pools, open pits for plastering and clay mining, vehicle ruts and hoof prints were the most preferred habitats (in terms of occurrence and abundance) for anopheline larvae. These habitats were either man-made or associated with anthropogenic activities. It should be noted that although many of these habitats, and especially hoof prints, are very small, they are very abundant in the landscape. Increasing human population in the catchment resulted in increased anthropogenic activities including deforestation, agricultural expansion, livestock rearing and brick making which could create suitable habitats for mosquito larvae [6,59]. Clearing and drainage, often for agricultural expansion creates favorable habitats for mosquitoes, thereby increasing malaria transmission [58,60]. In addition, agriculture can cause increased sedimentation due to erosion, which can slow or block streams and decrease the water depth, creating shallow waters ideal for mosquito breeding [59]. Earth excavation for brick making, pot making and pits dug for wall plastering provide a large number of mosquito larval habitats. In this study area, brick making activities were carried out in natural wetlands, where clay soil was used for brick making. In addition to creating mosquito breeding habitats, brick making is also considered as an important cause of deforestation, as it uses a huge amount of fire wood from wetland riparian forests. Deforestation may in turn alter the local microclimate and biodiversity [61], which in turn influences the distribution of malaria vectors.

Anopheline larvae were more abundant in small temporary habitats exposed to sunlight with low emergent plant and canopy cover. Emergent plants and/or canopy cover reduces the amount of sunlight reaching the aquatic habitats, thereby reducing water temperature [17]. Low water temperature causes a decline in microbial growth upon which mosquito larvae feed [17]. Smaller water bodies are generally characterized by high water temperature, which eventually led to rapid larval development time [62].

In this study, anopheline larvae occurred less frequently and were found at lower abundance in permanent habitats such as ponds, stream margins and natural wetlands. These habitats are home to a wide diversity of vertebrate and invertebrate predators and competitors and their presence is likely suppresses the density of mosquito larvae [63]. Several studies pointed out that aquatic insects belonging to the orders Coleoptera, Odonata and Hemiptera are responsible for significant reductions in mosquito populations and could be considered in integrated vector management programs [1]. Predators reduce the abundance of mosquito larvae directly via predation, avoidance of oviposition or indirectly via competition for food resources [64]. Some predators (especially those with chewing mouthparts) eat their prey (Odonata), but others suck the body fluid (hemolymph) of the prey (many beetle larvae and Hemiptera) [1]. Some species of mosquito larvae reduce the chance of predator detection by reducing their activities [65,66]. However, this has the disadvantage of reducing feeding efficiency, which in turn prolongs larval development and is also likely to result in smaller adults with probably a reduced longevity and fecundity [65].

Previous studies have reported that the occurrence and abundance of mosquito larvae reduced in response to predator cues [67]. For example, backswimmers (Notonectidae) released predator cues (kairomone) that have a potency to repel ovipositing female mosquito over a week [1]. The predator’s cues not only affect mosquito oviposition, but also cause a decrease in mosquito survival, delayed immature development and reduction in body size of emerged mosquitoes [1,67]. The abundance of anopheline larvae can be limited by the presence of competitors in permanent habitats (e.g. natural wetlands). Molluscs and anurans are the most common competitors, which feed on the same type of food as mosquito larvae. Several studies have shown that competitors decrease mosquito longevity and increase the developmental time of mosquito larvae [1]. In this study, Box- and Whisker-plots showed that permanent habitats support a significantly higher abundance of macroinvertebrate predators and competitors than semi-permanent and temporary habitats (Figure 8). The conditional analysis and ordination diagram demonstrated that the abundance of anopheline larvae was negatively related to invertebrate predators.

The decision tree models, redundancy (RDA) analysis and the GLMs indicated that both biotic and abiotic environmental factors influence the abundance and distribution of anopheline larvae. Our results indicate that preferred (in terms occurrence and abundance) anopheline breeding sites were temporary habitats, most notably, pits for plastering and clay mining, agricultural trenches, rain pools, vehicle ruts and small natural sunlit temporary breeding habitats such as animal hoof prints and rain pools. The overall suitability of these temporary habitats was mainly influenced by water temperature, vegetation cover, and presence of predators and competitors.

Permanent habitats such as natural wetlands in the vicinity of Jimma town were less suitable as breeding sites for anopheline larvae (Figure 3). This may be due to the high abundance and diversity of non-mosquito invertebrates and fish in these habitats (Figure 8), which could suppress mosquito population by predation and competition. This suggests that conservation of permanent habitats such as natural wetlands could be one strategy in the integrated malaria control program. The use of predaceous insects to control mosquito larvae is not only ecologically friendly but also a means by which more effective and sustainable control can be achieved [1]. However, detailed knowledge on the interaction between mosquito larvae and their predators is crucial for implementing successful vector control interventions. Contrarily, environmental modifications (e.g. drainage) of permanent habitats such as natural wetlands for malaria control could reduce the natural predator and competitor population densities, and thus be counter-productive and enhance the occurrence and abundance of mosquito larvae.

The findings of this study suggest that malaria vector control intervention strategies in the study area should target (man-made) temporary water bodies. In view of the presence of insecticide resistant anopheline mosquito populations in the study area, targeting these temporary water bodies for anopheline mosquito larval control should be considered as an alternative to reduce vector density and hence prevalence and/or incidence of malaria at a local scale. The use of microbial insecticides such as *Bacillus thuringiensis* can be more environmentally friendly in natural systems [68]. However, the use of chemical insecticides in natural systems may pose deleterious effects on non-target organisms such as predators and competitors. The relationships found in this study between anopheline larvae and biotic and abiotic variables are mainly valid for the most common species *Anopheles arabiensis* found in the region [22]. The main limitation of the present study is that the results may be applicable to some areas where the same or similar species predominate, but not to the other areas with different species. Therefore, it would be interesting to further investigate whether these relationships can be generalized for other regions and different species.

## Conclusions

The findings of this study revealed that anopheline larvae occurred frequently and were more abundant in shallow temporary habitats. Their abundance is positively influenced by high water temperature and the absence of natural predators and competitors. Malaria vector control intervention strategies should target these temporary water bodies in order to optimize the efficacy of malaria control. The drainage or conversion of natural marshlands for larval control may not be an efficient vector control strategy as wetlands were not found to be the most prolific mosquito breeding sites in the study area.

## Competing interests

The authors declare that they have no competing interests.

## Authors’ contributions

STM, DY, LD, NS conceived the ideas. STM, DY, AA, WL, collected the data. STM, PB, LDM and PG performed the data analysis. STM, DY, SV and PB led the writing. All authors read and approved the final manuscript.

## Supplementary Material

Additional file 1 Annex 1 Frequency of Macroinvertebrate families collected in the surveyed sites with functional feeding group. Click here for file

Additional file 2 Annex 2 Method description of Generalized Linear Models and output logistic regression model. Click here for file

Additional file 3 Annex 3 Output negative binomial model. Click here for file
