# Supplementary material for: Physico-chemical and biological characterization of anopheline mosquito larval habitats (Diptera: Culicidae): implications for malaria control
Source: Parasit Vectors. 2013 Nov 4;6:320. doi: 10.1186/1756-3305-6-320 (PMC4029358; doi:10.1186/1756-3305-6-320)
Supplement: Additional file 1 — Annex 1 Frequency of Macroinvertebrate families collected in the surveyed sites with functional feeding group. [file 1756-3305-6-320-S1.docx]

**Annex 1. Frequency of Macroinvertebrate families collected in the surveyed sites with functional feeding group.**

| Family | Frequency of occurrence (%) | Functional feeding group | Reference |
| --- | --- | --- | --- |
| Aeshnidae | 12 | Predator | Bode et al., 1996 |
| Baetidae | 27 | Gatherer collector | Hauer and Lamberti, 1996 |
| Belostomatidae | 24 | Predator | Lunde and Resh, 2010 |
| Brachycentridae | 1 | Gatherer collector & Shredder | Bode et al., 1996 |
| Caenidae | 15 | Gatherer collector | Hauer and Lamberti, 1996 |
| Ceratopogonidae | 7 | Predator | Hauer and Lamberti, 1996 |
| Chaoboridae | 1 | Predator | Bode et al., 1996 |
| Chironomidae | 37 | Gatherer collector | Bode et al., 1996 |
| Chrysomelidae | 4 | Shredder | Hauer and Lamberti, 1996 |
| Coenagrionidae | 37 | Predator | Hauer and Lamberti, 1996 |
| Corduliidae | 8 | Predator | Bode et al., 1996 |
| Corixidae | 45 | Predator | Barbour et al., 1999 |
| Culicine | 28 | Filterer-collector | Bode et al., 1996 |
| Dixidae | 5 | Gatherer collector | Bode et al., 1996 |
| Dytiscidae | 37 | Predator | Bode et al., 1996 |
| Elmidae | 8 | Shredder & Scraper | Hauer and Lamberti, 1996 |
| Ephermerllidae | 1 | Gatherer collector | Bode et al., 1996 |
| Erpobdelidae | 9 | Predator/parasite | Lunde and Resh, 2010 |
| Gerridae | 6 | Predator | Lunde and Resh, 2010 |
| Glossosomatidae | 1 | Scraper | Hauer and Lamberti, 1996 |
| Glossiphoniidae | 21 | Predator | Bode et al., 1996 |
| Gomphidae | 1 | Predator | Bode et al., 1996 |
| Gyrinidae | 16 | Predator | Bode et al., 1996 |
| Helodidae | 10 | Scraper | Bode et al.,1996 |
| Heptageniidae | 3 | Scraper & Gatherer collector | Hauer and Lamberti, 1996 |
| Hirudinidae | 1 | Predator | Hauer and Lamberti, 1996 |
| Hydrobilidae | 1 | Scraper | Barbour et al., 1996 |
| Hydrometridae | 5 | Predator |  |
| Hydrophilidae | 38 | Gatherer collector | Bode et al., 1996 |
| Hydropsychidae | 4 | Filterer-collector | Hauer and Lamberti, 1996 |
| Lepidostomatidae | 1 | Shredder | Hauer and Lamberti, 1996 |
| Leptoceridae | 3 | Shredder | Hauer and Lamberti, 1996 |
| Libellulidae | 30 | Predator | Hauer and Lamberti, 1996 |
| limnephilidae | 1 | Shredder | Bode et al., 1996 |
| Lymnaeidae | 16 | Scraper | Barbour et al., 1996 |
| Mesoveliidae | 1 | Predator | Lunde and Resh,2010 |
| Naididae | 4 | Gatherer collector /predator | Bode et al., 1996 |
| Naucoridae | 7 | Predator | Hauer and Lamberti, 1996 |
| Nepidae | 28 | predator | Hauer and Lamberti, 1996 |
| Notonectidae | 13 | Predator | Lunde and Resh,2010 |
| Physidae | 3 | Scraper | Barbour et al., 1996 |
| Piscicolidae | 10 | unkown |  |
| Planorbidae | 2 | Scraper | Barbour et al., 1996 |
| Psychodidae | 8 | Gatherer collector | Hauer and Lamberti, 1996 |
| Simuliidae | 7 | Filterer-collector | Bode et al., 1996 |
| Sphaeriidae | 1 | Filterer-collector | Barbour et al., 1996 |
| Stratiomyidae | 1 | Gatherer collector | Bode et al., 1996 |
| Syrphidae | 6 | Gatherer collector | Hauer and Lamberti, 1996 |
| Tipulidae | 1 | Shredder, Gatherer collector & predator | Hauer and Lamberti, 1996 |
| Tubificidae | 2 | Gatherer collector | Barbour et al., 1999 |
| Veliidae | 4 | Predator | Bode et al., 1996 |
| Tadpole | 45 | Filterer-collector and predator | Alting et al., 2007 |

**References**

Alting, R., Whiles, M, Taylor, C.L., 2007. **What do tadpoles really eat? Assessing the trophic status of an understudied and imperiled group of consumers in freshwater habitats.** Freshwater Biology 52, 386–395.

Barbour MT, Gerritsen J, Griffith GE, Frydenborg R, McCarron E, White JS, Bastian ML: **A framework for biological criteria for Florida streams using benthic macroinvertebrates.** J N Am Benthol Soc 1996, **15**:185–211.

Barbour MT, Gerritsen J, Snyder BD, Stribling JB: **Rapid Bioassessment Protocols for Use in Streams and wadeable Rivers: Periphyton, Benthic Macroinvertebrates and Fish.** Second Edition. U.S. Environmental Protection Agency, Office of Water, Washington, DC. 1999.

Bode RW, Novak MA, Abele LA: **Quality assurance work plan for biological stream monitoring in New York State.** NYS Department of Environmental Protection, Division of Water, Bureau of Monitoring and Assessment, Stream Biomonitoring Unit, Albany, NY, 1996.

Hauer F, Lamberti G: **Methods in stream ecology.** Academic Press, New York, New York, USA. pp. 674, 1996.

Lunde KB, Resh VH: **Development and validation of a macroinvertebrate index of biotic integrity (IBI) for assessing urban impacts to Northern California freshwater wetlands.** Environ Monit Assess 2011, 184:3653-3674.
