# Supplementary material for: Physico-chemical and biological characterization of anopheline mosquito larval habitats (Diptera: Culicidae): implications for malaria control
Source: Parasit Vectors. 2013 Nov 4;6:320. doi: 10.1186/1756-3305-6-320 (PMC4029358; doi:10.1186/1756-3305-6-320)
Supplement: Additional file 2 — Annex 2 Method description of Generalized Linear Models and output logistic regression model. [file 1756-3305-6-320-S2.doc]

**Annex 2. Method description of Generalized Linear Models and output logistic regression model**

Method description of Generalized Linear Models

Generalized linear models (GLMs) are mathematical extensions of linear models that provide a less restrictive form than classic multiple regressions by providing error distribution for the dependent variable other than normal and non-constant variance functions. They are also based on an assumed relationship called a link function between the mean of the response variable and the linear combination of the predictor variables (Zuur et al. 2009).

Generalized linear models were developed in R (version 2.15.1, The R Foundation for Statistical Computing, 2012) to determine which environmental (biotic and abiotic) variables significantly explained the occurrence and abundance of anopheline larvae. Prior to the modeling, we tested for collinearity among all predictor variables using Pearson correlation coefficient. If variables were highly correlated, one of both was removed (r > 0.7). Outliers were removed as well based on visual dot plots according to Zuur et al. (2009). We used logistic regression to model the occurrence of anopheline larvae. For the logistic regression, the response variable was transformed by the logit link function, which transforms bound probabilities (between 0 and 1) to unbound values (Zuur et al. 2009). Similarly, Poisson regression (log link function) was used to model the abundance of anopheline larvae. We started with a full model including all variables without interactions. The forward–backward stepwise model selection method using Akaike’s information criteria (AIC) was used to select the most parsimonious model. A lower AIC indicates a better model. Models were fitted using a maximum likelihood method (Zuur et al. 2009). Homogeneity was checked by plotting residuals of every model against its respective predictors.

References:

Zuur AF, Elena NI, Walker NJ, Saveliev AA, Smith GM: Mixed effects models and extensions in ecology with R. New York: Springer, 2009.

Main results of logistic regression model

Call:

Glm (formula = Anopheline presence ~ Habitat type + Permanency + Canopy cover (%) + Emerged plant cover + Invertebrate predator + Fish + Competitors, family = binomial, data = dataset mosquito)

**Table A2.1. Results of logistic regression analysis**

|  |  | **Parameter estimate** | **Std. Error** | **z value** | **Pr(>|z|)** |
| --- | --- | --- | --- | --- | --- |
| **Intercept** |  | 2.376e+00 | 1.043e+00 | 2.279 | 0.02267 * |
| **Habitat Type** |  |  |  |  |  |
|  | Marshland |  | Reference |  |  |
|  | Reservoir | 3.396e+00 | 1.226e+00 | 2.770 | 0.00561 ** |
|  | Stream margin | 6.252e-01 | 8.528e-01 | 0.733 | 0.46349 |
|  | Pond | 2.036e-02 | 1.002e+00 | 0.020 | 0.98378 |
|  | Farm ditch | 1.547e+00 | 1.419e+00 | 1.090 | 0.27575 |
|  | Pit | 1.211e+00 | 1.190e+00 | 1.018 | 0.30887 |
|  | Road puddle | -1.224e+00 | 1.391e+00 | -0.880 | 0.37870 |
|  | Hoof print | 6.517e-01 | 1.419e+00 | 0.459 | 0.64602 |
|  | Rain pool | 1.759e+01 | 1.865e+03 | 0.009 | 0.99248 |
| **Permanency** | Temporary |  |  |  |  |
|  | Semi-permanent |  | Reference |  |  |
|  | Permanent | 1.762e+00 | 1.284e+00 | 1.372 | 0.17018 |
|  |  | -2.873e+00 | 7.134e-01 | -4.027 | 5.6e-05 *** |
| **Canopy cover (%)** |  | -4.151e-02 | .539e-02 | -2.697 | 0.00699 ** |
| **Emergent plant cover** | |  |  |  |  |
|  | <10% |  | Reference |  |  |
|  | 10-35% | 1.814e+01 | 1.499e+03 | 0.012 | 0.99034 |
|  | 35-65% | -0.536e-01 | 8.956e-01 | -0.506 | 0.61256 |
|  | 65-90% | -2.879e+00 | 1.042e+00 | -2.762 | 0.00575 ** |
|  | >90% | -2.932e+00 | 1.012e+00 | -2.896 | 0.00378 ** |
| **Invertebrate predator (abundance)**  **Fish (presence/absence)**  **Competitor (abundance)** | | -1.640e-02 | 7.461e-03 | -2.199 | 0.02790 * |
|  |  | -1.421e+00 | 6.031e-01 | -2.356 | 0.01849 * |
|  |  | -1.292e-01 | 4.689e-02 | -2.756 | 0.00586 ** |

Significant codes: 0 ‘***’ 0.001 ‘**’ 0.01 ‘*’ 0.05 ‘.’ 0.1 ‘ ’ 1

(Dispersion parameter for binomial family taken to be 1)

Null deviance: 270.63 on 215 degrees of freedom

Residual deviance: 129.02 on 197 degrees of freedom

AIC: 167.02

Number of Fisher Scoring iterations: 17

**Table A2.2. Logistic Regression m**odel performance

|  | | **Model Likelihood ratio test** | | **Discrimination index** | | **Rank discrimination index** | |
| --- | --- | --- | --- | --- | --- | --- | --- |
| **Observation** | 216 | LR chi^2^ | 141.60 | R^2^ | 0.673 | c | 0.938 |
| **0** | 69 | df | 18 | g | 4.215 | Dxy | 0.877 |
| **1** | 147 | Pr(> chi^2^ | <0.0001 | gr | 67.689 | gamma | 0.877 |
| **max |deriv|** | 0.1 |  |  | gp | 0.38 | tau-a | 0.383 |
|  |  |  |  | Brier | 0.090 |  |  |


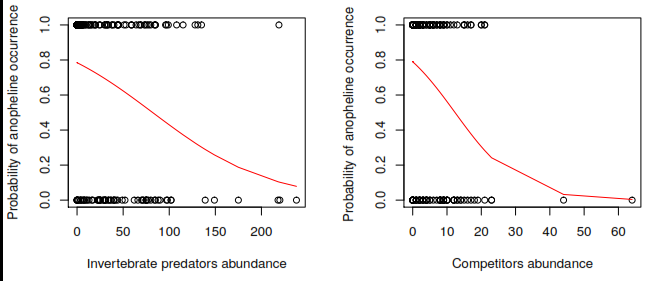


a)

b)

**Figure A2.1 Logistic regression model predicting the probability of occurrence of anopheline larvae as a function of the abundance of invertebrate predators (a) and competitors (b).**
