# Supplementary material for: Physico-chemical and biological characterization of anopheline mosquito larval habitats (Diptera: Culicidae): implications for malaria control
Source: Parasit Vectors. 2013 Nov 4;6:320. doi: 10.1186/1756-3305-6-320 (PMC4029358; doi:10.1186/1756-3305-6-320)
Supplement: Additional file 3 — Annex 3 Output negative binomial model. [file 1756-3305-6-320-S3.doc]

**Annex 3. Output negative binomial model**

Negative binomial model:

glm.nb (formula = Anopheline ~ Habitat type + Permanency + Canopy cover... + Emerged plants + Submerged plants + Water temperature + Fish + Competitors + offset(Number of dips), data = dataset mosquito, link = "log", init.theta = 1.314419314)

**Table A3.1. Results of negative binomial regression analysis**

|  |  | **Parameter estimate** | **Std. Error** | **z value** | **Pr(>|z|)** |
| --- | --- | --- | --- | --- | --- |
| **Intercept** |  | -2.789e+00 | 7.726e-01 | -3.610 | 0.00031*** |
| **Habitat type** | Reservoir | 1.155e+00 | 4.033e-01 | 2.864 | 0.004185 ** |
|  | Stream margin | 1.490e-01 | 3.408e-01 | 0.437 | 0.661974 |
|  | Pond | 4.963e-01 | 3.477e-01 | 1.427 | 0.153450 |
|  | Farm ditch | 3.135e-01 | 3.965e-01 | 0.791 | 0.429094 |
|  | Pit | -3.538e-02 | 3.938e-01 | -0.090 | 0.928415 |
|  | Road puddle | 4.313e-01 | 5.126e-01 | 0.841 | 0.400084 |
|  | Hoof print | 1.868e+00 | 4.495e-01 | 4.157 | 3.3e-05 *** |
|  | Rain pool | 8.955e-02 | 4.405e-01 | 0.203 | 0.838899 |
| **Permanency** | Semi-permanent | 3.589e-01 | 3.557e-01 | 1.009 | 0.312935 |
|  | Permanent | -1.250e+00 | 2.490e-01 | -5.021 | 5.1e-07 *** |
| **Canopy cover (%)** |  | -1.830e-02 | 6.302e-03 | -2.904 | 0.003687 ** |
| **Emergent plant cover** | 10-35% | 3.225e-01 | 3.258e-01 | 0.990 | 0.322269 |
|  | 35-65% | 2.602e-01 | 2.361e-01 | 1.102 | 0.270540 |
|  | 65-90% | 7.732e-01 | 2.407e-01 | 3.213 | 0.001313 ** |
|  | >90% | 9.025e-01 | 2.743e-01 | 3.290 | 0.001003 ** |
| **Submerged plants** | 10-35% | 3.225e-01 | 3.258e-01 | 0.990 | 0.322269 |
|  | 35-65% | 2.602e-01 | 2.361e-01 | 1.102 | 0.270540 |
|  | 65-90% | 7.732e-01 | 2.407e-01 | 3.213 | 0.001313 ** |
|  | >90% | 9.025e-01 | 2.743e-01 | 3.290 | 0.001003 ** |
| **Water temperature** |  | 1.029e-01 | 2.661e-02 | 3.867 | 0.00011 *** |
| **Fish (presence/absence)** |  | -5.567e-0 | 2.180e-01 | -2.554 | 0.010659 * |
| **Competitor (abundance)** | | -1.104e-01 | 2.503e-02 | -4.413 | 1.0e-05 *** |

Significant codes: 0 ‘***’ 0.001 ‘**’ 0.01 ‘*’ 0.05 ‘.’ 0.1 ‘ ’ 1

(Dispersion parameter for Negative binomial (1.3144) family taken to be 1)

Null deviance: 529.12 on 219 degrees of freedom

Residual deviance: 241.73 on 198 degrees of freedom

AIC: 1109.2

Number of Fisher Scoring iterations: 1

Theta: 1.314

Std. Err.: 0.192


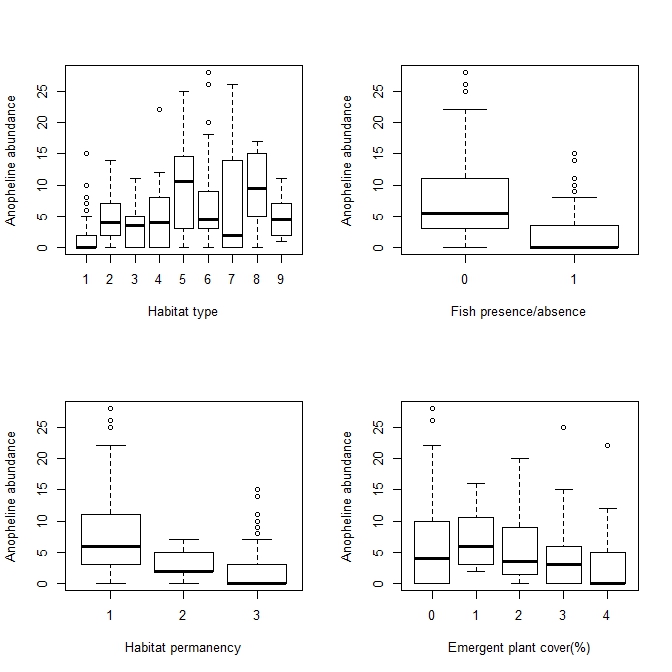


a)

b)

c)

d)

**Figure A3.1. Box plots showing the effect of habitat type (a), occurrence of fish (b), habitat permanency (c) and vegetation cover (d) on the abundance of anopheline larvae. Small black squares represent median numbers, boxes represent inter-quartile ranges (25–75% percentiles) and range bars show maximum and minimum values, circles are used to denote outliers. Habitat type (1= Marshland, 2 = Reservoir, 3 = Stream margin, 4 = Pond, 5 = Farm ditch, 6 = Pits, 7 = Road puddle, 8 = Hoof print, 9 = rain pool); Fish (0 = absence, 1 = present); Habitat permanency (1 = temporary, 2 = Semi-permanent, 3 = permanent); Emergent plant cover (0 = <10%, 1 = 10-35%, 2 = 35-65%, 3 = 65-90% , 4 = >90%).**

a)

b)


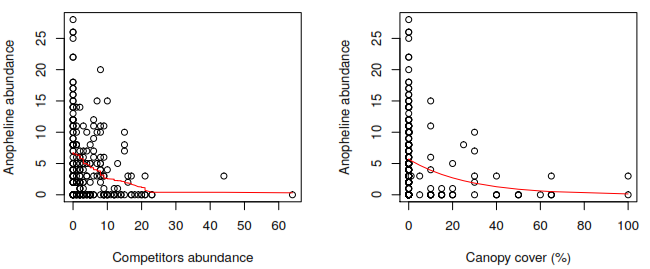


a)

b)


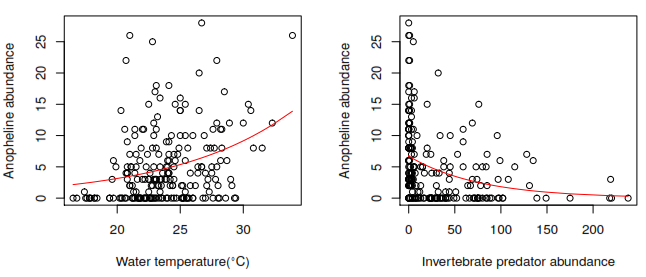


c)

**Figure A3.2. Negative binomial regression models predicting the abundance of anopheline larvae in function of competitor’s abundance (a), canopy cover (b), water temperature (c).**
